# In this month’s Bulletin

**DOI:** 10.2471/BLT.22.000722

**Published:** 2022-07-01

In the editorial section, Kidist Kebede Bartolomeos (414) makes the case for violence prevention. Matthew Myers Griffith et al. (415) argue for more investment in applied epidemiology.

Lynn Eaton (418–419) reports on collaboration needed to ensure water, sanitation and hygiene in humanitarian emergencies. Andrei Shukshin talks to Gulnaz Azhymambetova (420–421) about empowering nurses to deliver better patient care in Kyrgyzstan.

## Democratic Republic of the Congo 

### Where do child deaths occur?

Mattias Schedwin et al. (422–435) analyse survey and conflict data by province. 

## Democratic Republic of the Congo, Guinea, Liberia, Sierra Leone

### How to diagnose Ebola virus infections

Basilua Andre Muzembo et al. (447–458) review the evidence for test performance.

## India 

### Tracking obstetric emergencies

Divya Mecheril Balachandran et al. (436–446) assess indicators for near miss and maternal deaths.

## Pakistan

### Ending violence against women and girls

Azza Sarfraz et al. (462–464) call for a health-sector response to gender-based violence.

## Global

### Child health beyond survival 

Bolajoko O Olusanya et al. (459–461) describe an inclusive development and education agenda.

